# The effects of neuromuscular exercise on medial knee joint load post-arthroscopic partial medial meniscectomy: ‘SCOPEX’ a randomised control trial protocol

**DOI:** 10.1186/1471-2474-13-233

**Published:** 2012-11-27

**Authors:** Michelle Hall, Rana S Hinman, Tim V Wrigley, Ewa M Roos, Paul W Hodges, Margaret Staples, Kim L Bennell

**Affiliations:** 1 The University of Melbourne, Centre for Health, Exercise and Sports Medicine, Department of Physiotherapy, School of Health Sciences, Melbourne, VIC, 3010, Australia; 2 The University of Southern Denmark, Institute of Sports Science and Clinical Biomechanics, Odense, Denmark; 3 School of Health and Rehabilitation Sciences, University of Queensland, Brisbane, QLD, Australia; 4 Epidemiology & Preventive Medicine, Monash Medical School Building, The Alfred Hospital, Melbourne, VIC, Australia

## Abstract

**Background:**

Meniscectomy is a risk factor for knee osteoarthritis, with increased medial joint loading a likely contributor to the development and progression of knee osteoarthritis in this group. Therefore, post-surgical rehabilitation or interventions that reduce medial knee joint loading have the potential to reduce the risk of developing or progressing osteoarthritis. The primary purpose of this randomised, assessor-blind controlled trial is to determine the effects of a home-based, physiotherapist-supervised neuromuscular exercise program on medial knee joint load during functional tasks in people who have recently undergone a partial medial meniscectomy.

**Methods/design:**

62 people aged 30–50 years who have undergone an arthroscopic partial medial meniscectomy within the previous 3 to 12 months will be recruited and randomly assigned to a neuromuscular exercise or control group using concealed allocation. The neuromuscular exercise group will attend 8 supervised exercise sessions with a physiotherapist and will perform 6 exercises at home, at least 3 times per week for 12 weeks. The control group will not receive the neuromuscular training program. Blinded assessment will be performed at baseline and immediately following the 12-week intervention. The primary outcomes are change in the peak external knee adduction moment measured by 3-dimensional analysis during normal paced walking and one-leg rise. Secondary outcomes include the change in peak external knee adduction moment during fast pace walking and one-leg hop and change in the knee adduction moment impulse during walking, one-leg rise and one-leg hop, knee and hip muscle strength, electromyographic muscle activation patterns, objective measures of physical function, as well as self-reported measures of physical function and symptoms and additional biomechanical parameters.

**Discussion:**

The findings from this trial will provide evidence regarding the effect of a home-based, physiotherapist-supervised neuromuscular exercise program on medial knee joint load during various tasks in people with a partial medial meniscectomy. If shown to reduce the knee adduction moment, neuromuscular exercise has the potential to prevent the onset of osteoarthritis or slow its progression in those with early disease.

**Trial Registration:**

Australian New Zealand Clinical Trials Registry reference: ACTRN12612000542897

## Background

Osteoarthritis (OA) is a common musculoskeletal disease that is considered by the World Health Organisation as one of the ten leading causes of disease burden in high-income countries [1]. The knee is the most commonly affected lower limb joint [2]. Osteoarthritis is associated with pain, reduced physical function and high economic costs [3-5]. Given there is no cure for OA, it is widely recognised that OA prevention, particularly targeting groups at greater risk of developing the condition, is an important research priority to reduce the personal and societal disease burden [6]. Individuals who have undergone an arthroscopic partial meniscectomy (APM), a common surgical procedure for meniscal injury particularly involving the medial meniscus [7], constitute one such group at risk of tibiofemoral knee OA [8]. Meniscal tears can be categorised as traumatic or degenerative tears. Traumatic tears are typically longitudinal tears and result from an acute sports-related injury in young active individuals [9,10]. Alternatively, degenerative tears are generally observed as horizontal-cleavage lesions or flap tears [11] in middle-aged and older populations [10]. These degenerative tears are suggested as the first sign of knee OA [12]. In fact, it is estimated that 50% of individuals have radiographic signs of knee OA within 10–15 years following meniscectomy [12-14]. Moreover, accumulating magnetic resonance imaging (MRI) data suggest that structural changes begin soon after APM. Studies have observed an increase in cartilage loss over 2 years following APM [15], and a greater prevalence of cartilage defects between 3 months and 4 years post-APM [16] as compared to healthy controls. Furthermore, medial femoral cartilage integrity as measured by glycosaminoglycan (GAG) content was impaired 4 years following medial APM when compared to the reference lateral femoral condyle cartilage [17]. These findings highlight the urgent need to develop and evaluate rehabilitative interventions that may reduce the likelihood of OA development in this patient group.

The onset of knee OA following APM may be partly related to a higher distribution of medial knee joint load. The external knee adduction moment (KAM), is a widely used biomechanical marker of medial knee load distribution measured via three-dimensional gait analysis. Indeed a large cross-sectional study reported that individuals 3-months post-APM surgery had ~18% higher peak KAM bilaterally as compared to healthy controls during walking [18]. This highlights the greater medial distribution of knee joint load during walking in patients’ post-APM. The finding of higher medial joint load distribution post-APM is relevant to the pathogenesis of knee OA. The peak KAM is generally higher in those with established structural medial knee OA than in healthy controls [19-22] and also correlates with knee OA severity as measured on radiographs [21,23]. Furthermore, longitudinal studies have associated the peak KAM with the development of chronic knee pain in older people [24] as well as an increased risk of radiographic OA progression in those with established disease [25]. A greater KAM impulse, which takes into account the average magnitude and duration of medial loading throughout stance and may be a more sensitive marker of medial joint dynamic loading than peak KAM [26], has also been related to cartilage degradation in people following APM [27] and in those with knee OA [28]. Thus, it is apparent that medial knee joint loading (as inferred by KAM parameters) is associated with the pathogenesis of knee OA and if reduced, could potentially prevent or delay OA-initiation and progression in people following APM of the medial meniscus.

Exercise may be an effective strategy to reduce the greater medial knee joint load distribution observed post-APM. Exercise is recommended as part of rehabilitation post-meniscal surgery [29] and is also an attractive rehabilitation option considering that it is relatively safe, is a patient-driven strategy that can facilitate long-term self-management, is inexpensive and is clinically advocated in knee OA management [30,31]. There is limited evidence as to the effects of different exercise types on knee joint loading. Studies have examined the effects of knee and hip muscle strengthening [32-34] on medial knee joint loading in older patients with established symptomatic and radiographic knee OA. Despite improving OA-associated symptoms, no change in medial knee joint loading was found during walking with the tested strengthening programs [33,34]. However, some pilot evidence suggests that neuromuscular exercise may reduce loading across the knee joint in people with and without knee OA during more functionally challenging tasks than walking [35,36].

Neuromuscular training is a broad term used to incorporate many components of exercise training including: balance, perturbation, agility, plyometrics, strength and endurance [37,38]. Furthermore, neuromuscular training programs are designed with various aims: to prevent sports injuries in young athletes [39], to rehabilitate individuals post-sport related injuries [40] and more recently to prevent OA progression [37]. Therefore it is important to acknowledge that a vast array of exercises and intensities are considered neuromuscular in nature. However, regardless of the target population or aim of the training program, a common feature of neuromuscular training is the use of functional weight-bearing exercise positions. Physiologically, neuromuscular training aims to enhance the unconscious motor response by calling upon both afferent signal and central mechanisms responsible for dynamic control [41]. A randomised controlled trial (RCT) reported that following a 4-month neuromuscular training program, participants post-APM improved one-leg hop for distance and improved self-reported outcomes [17,42]. Importantly, this RCT also found improved cartilage quality as indicated by the GAG content on MRI following the neuromuscular exercise program [43]. Neuromuscular training has been found to reduce external abduction knee loads in young healthy females while landing from a jump [35] adding support to the premise that neuromuscular exercise can alter frontal plane moments. Of particular relevance to the current study is evidence that peak KAM can be reduced with neuromuscular exercise in middle-aged individuals with moderate levels of knee OA [36]. This uncontrolled pilot study included 13 individuals and found a 14% reduction in peak KAM during a one-leg rise task with no difference in peak KAM during walking following the 8-week training period. Despite the difference in the purposes of the neuromuscular exercise training programs [35,36], these findings from healthy and OA cohorts suggest that neuromuscular training has potential to reduce frontal plane knee moments. Furthermore, these studies suggest that potential alterations in knee joint load distribution appear to be detected during more challenging motor tasks following neuromuscular training. This hypothesis is further supported by recent cross-sectional evidence in APM individuals describing altered neuromuscular function during a forward lunge [44] that were not detected during stair descent when comparing the operated and non-operated legs [45].

Given that neuromuscular training has the potential to alter knee joint load, we hypothesise that neuromuscular training may be an effective load-modifying intervention to rehabilitate individuals post-APM, a patient group at risk of developing knee OA. However to date, this has not been evaluated. The primary objective of this study (‘SCOPEX’) is to determine whether a neuromuscular exercise program (‘ALIGN’) reduces medial knee joint loading in people with a recent medial APM, as measured by the peak external KAM during normal pace walking and one-leg rise. The secondary objectives are to evaluate the effect of the ALIGN program post-medial APM on: i) the peak external KAM during fast pace walking and one-leg hop for distance; ii) the external KAM impulse during: walking (normal pace and fast pace), one-leg rise and one-leg hop for distance; iii) knee and hip muscle strength; iv) electromyographic muscle activation patterns; v) functional physical performance and vi) patient-reported outcomes knee pain, other symptoms, activities of daily living, sport and recreation and knee-related quality of life.

### Primary hypothesis

The 12-week neuromuscular exercise program will reduce medial knee joint loading, as characterised by the peak external KAM during normal pace walking and one-leg rise, when compared to a control group not receiving the neuromuscular exercise program.

### Secondary hypotheses

The 12-week neuromuscular exercise program will: i) reduce the peak external KAM during fast-pace walking and one-leg hop for distance; ii) reduce the external KAM impulse during walking (normal and fast pace), one-leg rise and one-leg hop for distance; iii) improve knee and hip muscle strength; iv) alter electromyographic muscle activation patterns; v) improve functional physical performance; vi) improve self-reported outcomes (knee pain, other symptoms, activities of daily living, function, sport and recreation function and knee-related quality of life) when compared to a control group.

## Methods

### Trial design

**Figure 1:**
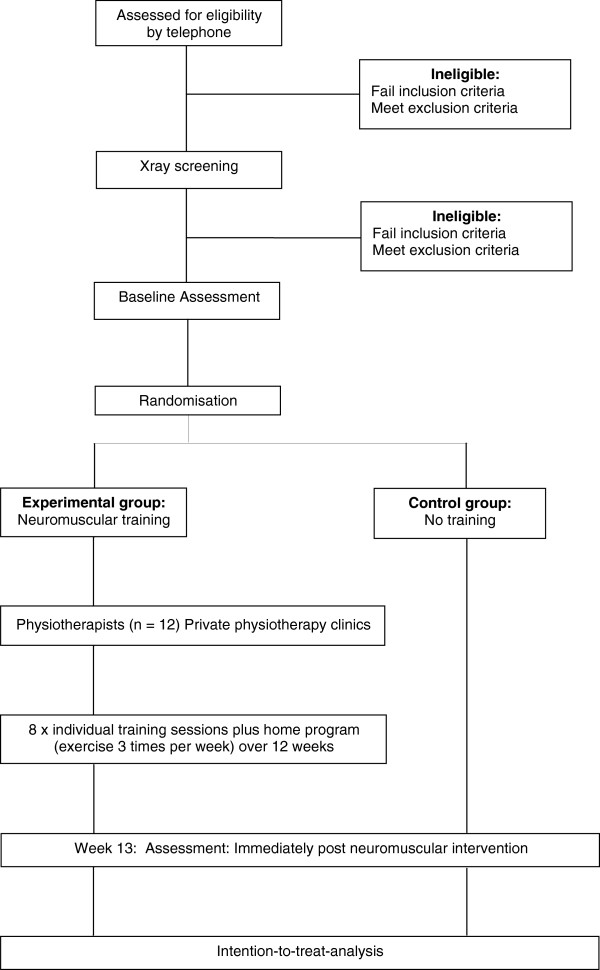
**Trial Protocol** .

This will be an assessor-blinded, randomised controlled trial of a 12-week physiotherapist-supervised and home-based neuromuscular exercise intervention. Assessment will be performed at baseline and immediately following the intervention (12 weeks). The protocol conforms to CONSORT guidelines for non-pharmacological studies [46] (Figure 1) and has been registered with the Australia and New Zealand Clinical Trials Registry prior to study commencement.

### Participants

Sixty-two participants with a recent isolated medial APM will be recruited from metropolitan Melbourne, Australia. The primary recruitment strategy will be via screening of surgical records of several Melbourne-based orthopaedic surgeons. Additionally, we will recruit individuals by advertising through University staff newsletters and Facebook. To be eligible, participants must be aged between 30–50 years and have undergone an isolated medial APM in the past 3–12 months.

The exclusion criteria are:

i) an average overall pain severity ≥3 out of 10 on an 11 point numerical rating scale in the past week

ii) signs of moderate to severe radiographic knee OA (Kellgren-Lawrence grade >2) [47]

iii) full tears of the anterior or posterior cruciate knee ligaments

iv) anterior or posterior ligament reconstructions

v) spinal or lower extremity pain (other than the affected knee) that limits everyday activities or required treatment in the past month

vi) high levels of physical activity defined as participation in an organised team or individual sport that requires regular competition and requires some form of systematic training [48]

vii) history of lower limb surgery (other than knee arthroscopy)

viii) other form of arthritis, diabetes, cardiac circulatory conditions that limit everyday activities (as determined by the Self-administered Co-morbidity Questionnaire)[49]

ix) neurological condition such as Parkinson’s disease, multiple sclerosis or stroke

x) any condition precluding safe participation in exercise

(xi) unable to complete the study protocol

(xii) inadequate written or spoken English

### Procedure

Individuals who meet the inclusion criteria will be sent information in the mail. Approximately two weeks later these individuals will be contacted by telephone and invited to undergo further screening for exclusion criteria. Screening records will be maintained to detail the criteria eliminating individuals found to be ineligible. Following further screening for exclusion criteria via telephone, people who are potentially eligible will undergo a semi-flexed standing postero-anterior x-ray of their partially meniscectomised knee at one of three trial radiology centres (unless they can provide their own radiographs taken within the past 12 months). The x-rays will be graded by one of two experienced clinicians. Eligible participants will attend the Centre for Health Exercise and Sports Medicine (CHESM) at The University of Melbourne for baseline and follow-up testing. Following baseline testing, participants will be randomised into one of two groups: i) neuromuscular exercise training; or (ii) no neuromuscular exercise training. The 12-week neuromuscular exercise training intervention will include 8 individual physiotherapist visits: twice in the first week, one in each of the second and third weeks and fortnightly thereafter. Participants in this group will also perform a home-based exercise program throughout the 12-week intervention. All participants will provide written informed consent.

### Blinding

Baseline and follow-up assessments will be performed by the same outcome assessor who will remain blinded to group allocation and who will not be involved in providing the neuromuscular exercise program. Furthermore, the outcome assessor will not visit any of the physiotherapist centres. Participants will be instructed not to disclose details about their group allocation to the blinded assessor. By virtue of the design, it is not possible to blind the participants or the physiotherapists.

### Randomisation and allocation concealment

A researcher with no other involvement in the study will prepare the randomisation schedule *a priori* using a computer generated random numbers table. Randomisation will be conducted by random permuted blocks of size 6 to 8 and stratified by gender. To conceal randomisation, the same independent researcher will prepare consecutively numbered, sealed opaque envelopes. The envelopes will be kept in a locked location and only accessible to the independent researcher responsible for randomising participants following baseline testing. Another independent researcher will then schedule the first two physiotherapist appointments for those in the neuromuscular exercise group.

### Intervention

Participants will be asked to refrain from seeking other forms of treatment during the trial. Additionally, participants from both groups will be asked to refrain from performing additional physical activity to what they currently perform. Analgesia will be permitted as required.

### Neuromuscular exercise group

Similar to previous neuromuscular exercise programs described for knee OA patients [37,38], our neuromuscular exercise program, ALIGN, is based on biomechanical and neuromuscular principles that aim to improve sensorimotor function. Considering that the context of the current study is OA prevention and delay of progression, participants will be instructed to focus neutral alignment of the hip, knee and foot during weight bearing positions. Furthermore, participants will also be encouraged to focus on the performance quality of each exercise. The neuromuscular exercise intervention, ALIGN will target muscles of both lower limbs. The exercises were selected based on previous research demonstrating these exercises or similar variations to have reduced medial knee joint load, improved cartilage quality and physical function in people at risk of developing or progressing established knee OA [36,42,43].

### Study physiotherapists

Twelve physiotherapists in private practices at multiple locations throughout metropolitan Melbourne, Australia will deliver the neuromuscular exercise program titled “ALIGN”. The physiotherapists have an average of 15 years (range 3–36 years) of clinical experience since qualification and 11 years (range 0–27) of post-graduate clinical musculoskeletal experience. Three (25%) have a postgraduate Masters qualification in sports or manipulative/musculoskeletal therapy. The physiotherapists will attend a 3-hour training session addressing the delivery of the neuromuscular exercise program and receive a detailed treatment manual. This large number of study physiotherapists is necessary for feasibility and to improve the generalisability of study findings. Physiotherapists will be supplied with equipment for their clinics to demonstrate the exercises to participants. Participants will choose a project physiotherapist to attend based on their geographical location and time availability. Training will commence within one week of baseline assessment.

### First physiotherapist visit

The initial visit with the physiotherapist will last 45 minutes, during which participants will be educated about the goals and benefits of the neuromuscular exercise program. Potential barriers to exercise will also be addressed. Individuals with knee OA have expressed that pain or the fear of pain prevents exercise participation [50]. Therefore, participants will use a pain monitoring scale to guide their home exercises, where zero represents “no pain” and ten represents “worst possible pain”. On this scale, exercising with a pain score up to two is considered ‘safe’, exercising with pain up to five is considered ‘acceptable’ and exercise when pain scores are above five is deemed ‘high risk’ [51]. However, due to the minimal levels of pain in this cohort, we do not anticipate that pain will be an issue. Nonetheless, it is thought that monitoring knee pain before and after each exercise session will give participants confidence to exercise. Another commonly expressed barrier to exercise is lack of time [52]. During the first session, physiotherapists will encourage participants to consider how they can best fit the neuromuscular exercise sessions into their weekly schedule. Following the initial education and discussion of exercise barriers, participants will be taught their individualised training program by the physiotherapist. The 6 core exercises in the ‘ALIGN’ program are described in Table 1. Physiotherapists will determine the appropriate starting level of each exercise for individual participants based on the quality of performance by the participant and the verbalised degree of difficulty from participants.

**Table 1:** Neuromuscular exercise program; ALIGN

**Exercise**	**Primary Muscles Targeted**	**Description of starting level**
**A**bdominal crunches	Abdominal muscles	Performed in supine with knees flexed on exercise ball, participants perform abdominal crunches
bridge	Hamstrings, gluteals, hip adductors	Performed in supine with knees flexed on the exercise ball, participants rise up and hold the position with a towel between knees
**L**unge	Gluteals, knee extensors and flexors	Standing upright, participants lunge forward and return to starting position
**I**ncomplete circle	Hip abductors	Performed in a unipedal stance while the opposite limb rotates in a semi-circular motion
**G**et tapping	Knee extensors and hip abductors	Performed in a unipedal stance on a stepper, as the opposite leg taps the ground behind the step
k**N**ee bends	Knee extensors, gluteals and hip abductors	Performed in a bipedal stance in front of a chair with feet hip width apart, knees are bend so the bottom nearly touches the chair or heels remain in full contact with the floor

### Home training sessions

Participants will be instructed to perform their individualised neuromuscular exercises at home three times per week in addition to exercises performed during scheduled supervised physiotherapy visits. Each session will last approximately 30–35 minutes, accumulating to a total of >90 mins of exercise per week. Despite there being no specific guidelines for neuromuscular training, based on the authors’ experience and the American College of Sports Medicine recommendations for neuromotor exercise, (2–3 days per week; 20–30 minutes each day) [53], it was considered that >90 minutes per week would be adequate for physiological adaptations.

### Follow-up physiotherapist sessions

During the subsequent seven physiotherapist sessions each lasting 30 minutes, physiotherapists will assess adherence to the home exercise program by reviewing the participants’ weekly logbook and inquiring about any adverse events experienced. Physiotherapists will then assess exercise technique and increase the exercise intensity (in terms of the level) as necessary. Exercise progression will be based on the physiotherapists’ assessment of the quality of the exercise performance and on the participant’s reported pain response and degree of difficulty experienced. Varying surfaces and weight will be used to increase the exercise difficulty. The 6 core exercises and respective levels of difficulty are detailed in an Additional File. During the period between physiotherapy visits, participants are also encouraged to progress by increasing the volume of the repetitions and sets. Participants will start performing 2 sets of 12 repetitions. Participants can progress their training by opting to increase the volume to 2 sets of 15 repetitions; 3 sets of 12 repetitions and 3 sets of 15 repetitions respectively. However, the level of difficulty is, as previously described, increased by the physiotherapist. Physiotherapists will maintain standardised treatment notes.

### Control group

Participants in the control group will not receive any interventions during the 12-week study period.

### Outcome measures

Baseline descriptive data including age, gender, date of surgery, surgical procedure performed, and the knee undergoing surgery will be obtained by questionnaire and from surgical records. Radiographic disease severity will be assessed from the screening x-ray using the Kellgren and Lawrence grading system [47]. Height and body mass will be measured. For a summary of all measures collected in the study see Table 2.

**Table 2:** Outcome measures summary

**Primary outcome measures**	
Peak external knee adduction moment during stance	a) Walking (normal pace)
	b) One-leg rise
**Secondary outcome measures**	
Peak external knee adduction moment during stance	a) Walking (fast pace)
	b) One-leg hop for distance
External knee adduction moment impulse during stance	a) Walking (normal pace and fast pace)
	b) One-leg rise
	c) One-leg hop for distance
Knee and hip muscle strength	Isometric and isokinetic knee extensors and flexors (isokinetic dynamometer)
	Isometric hip abductors and hip adductors (instrumented manual muscle tester)
Muscle activity patterns and co-contractions	Surface electromyography
Objective functional performance	a) One-leg rise
	b) Knee bends
	c) One-leg hop for distance
Pain, other symptoms, function in ADL and Sport and Recreation, quality of life	Knee injury and osteoarthritis outcome score
**Other measures**	
Peak external knee flexion moment	3-dimensional movement analysis
Dynamic tibial alignment, lateral trunk lean angle, internal foot rotation angle	3-dimensional movement analysis
Static alignment	Inclinometer
Disease severity	X-ray (baseline)
Physical activity levels	Lower Extremity Activity Scale
Adverse events	Participant log-book (follow-up)
Adherence to exercise program	Participant log-book (follow-up)
Treatment session attendance	Therapist treatment records (intervention group only) (follow-up)

All measures are obtained at baseline and 13 weeks, unless otherwise indicated.

### External knee adduction moment

Three-dimensional movement analysis will be used to assess participant’s movement as they perform three different tasks wearing standardised footwear (Dunlop Volley, Pacific Brands, Australia): i) walking (normal and fast pace), ii) one-leg rise and iii) one-leg hop for distance. Kinematic and kinetic data will be recorded using a 12-camera motion analysis system (Vicon MX, Oxford, UK) and three force plates (AMTI, MA, USA). Participants will be assessed while walking at two speeds: a normal pace walk described to participants as ‘the pace you would walk normally’ and a fast pace walk described as ‘the pace you would walk in a hurry’. Walking speed will be monitored, and six successful trials for normal and fast pace (defined as a complete foot strike on a single force plate) will be collected for each leg. At follow-up, if the average walking speed of the six trials for either normal pace or fast pace walking is outside a ±5% range from baseline walking speed, participants will be asked to adjust their walking speed accordingly. This is to ensure that six trials are collected within 5% of baseline walking speed.

For the one-leg rise task, participants will be seated in a standardised position with a chair height of 48cm, seat depth of 75% thigh length and 100° knee flexion. Participants will be instructed to flex the non-test knee holding the leg off the ground throughout the task, and rise from the seat using the test leg (leg being assessed) until standing upright, and then return to the seated position. If participants cannot rise from the seat, the chair height will be increased in increments of 2.5cm until the task can be achieved. Following two practice trials, three trials will be recorded and averaged for each leg. For the one-leg hop for distance task, participants will be instructed to stand on one foot with their arms across their chest and instructed to jump as far forward as possible and land steadily, without over-balancing on the same foot. Following, two practice trials at submaximal effort, three maximum trials where a complete foot lands on a single force plate will be averaged and collected for each leg. In an effort to minimise fatigue, three trials are considered adequate to assess the more demanding tasks (one-leg rise and one-leg hop). Reflective markers will be placed on the participant according to the University of Western Australia (UWA) marker set [54]. Data from the reflective markers (sample rate 120Hz) and ground reaction forces (sample rate 1200Hz) will be used to calculate external joint moments via inverse dynamics (Besier et al., 2003). The primary variables of interest are the peak external KAM (Nm/(BWxHT) %) and KAM impulse (Nm.s/(BWxHT) %), both normalised for body weight times height. The test-retest reliability of the UWA model for the external frontal plane moment curve during walking is 0.75 (coefficient of multiple determination, *r*^*2*^) [55]. Additional outcome measures that can influence the KAM during the stance phase will also be assessed, including frontal plane alignment of the tibia, peak external knee flexion moment, trunk lean in the frontal plane and internal foot rotation.

### Muscle strength

Isometric and isokinetic knee muscle strength and isometric hip abductor and adductor strength will be assessed on the previously operated leg. Maximal isometric quadriceps and hamstring strength will be measured at 60° knee flexion using a dynamometer (Humac NORM, CSMI, Massachusetts, USA). Participants will be secured in a standardised seated position. For knee strength assessment, participants will perform two sub-maximal efforts, followed by 3 maximal isometric contractions for 5 seconds each, and separated by 40 seconds rest. The maximum of 3 trials will be used for analysis. Following sub-maximal isokinetic practice efforts, maximal concentric isokinetic quadriceps and hamstring strength at 60°/sec will be measured with the participant performing 5 maximal efforts. The test-re test reliability for isometric knee strength in our laboratory for knee OA individuals is ICC 0.93.

Maximal isometric hip abductor and adductor torque will be measured using a hand-held dynamometer (Lafayette MMT 01163, Lafayette Instruments, IN, USA). For these hip strength assessments, participants will be positioned in supine with the study limb in slight abduction with no hip or knee flexion and with the pelvis and contralateral limb stabilised (Pua et al., 2008). The dynamometer will be placed over the lateral and medial femoral condyles for the hip abduction and adduction efforts, respectively. After a single sub-maximal attempt participants, will be instructed to perform two maximal trials each of 3 seconds duration, separated by 40 seconds of rest. For the hip strength assessments, the mean of two maximal trials will be used in the analysis. The reliability for hip abductor strength in our laboratory for hip OA individuals is excellent (ICC 0.84; 95% CI 0.55-0.94) [56]. For hip adductor strength, reliability in healthy individuals is ICC 0.78 (95% CI; 0.30-0.95) albeit, a slight variation on the method used in the current study [57]. All participants will receive strong standardised verbal encouragement to give their best effort during maximal trials. All strength data will be reported as torque normalised to body mass (Nm/kg).

### Muscle co-contraction and activation patterns

Muscle activity will be recorded during walking, one-leg rise and hop for distance trials using surface electromyography (EMG) sampled synchronously with kinematic data via a telemetered Noraxon Telemyo 900 system (Noraxon, AZ, USA). Surface electrodes will be placed unilaterally on the gluteus medius, quadriceps (vastus lateralis and medialis, rectus femoris), hamstrings (biceps femoris and semimembranosus) and medial and lateral gastrocnemius of the APM leg. EMG recordings during maximal isometric knee flexion, knee extension, hip abduction and plantar flexion will be used to normalise EMG data during walking, one-leg rise and hop for distance tasks. Total activation and relative activation of individual and grouped muscles will be investigated.

### Objective measures of function

Functional tests will be performed for each leg and will include the i) 30 second one-leg rise test; ii) 30-second knee bend test, and iii) one-leg hop for distance test. For the one-leg rise test, participants will sit in a standardised seated position (as described above). Participants will flex the non-test leg and rise from the seat using the test leg until standing upright and return to the seated position as quickly as possible. The number of successful, controlled one-leg rises from the chair during 30-seconds will be counted. The 30-second knee bend test assesses endurance of the hip and knee extensors. Participants will begin the test in a standardised position in single-leg standing, with the long axis of the foot aligned to a vertical line with toes placed perpendicularly. Participants will be instructed to bend the knee without bending at the hip, until the toes can no longer be seen and return to an upright position as quickly as possible [58]. The maximum number of complete repetitions performed in 30 seconds will be recorded. The one-leg hop test gives an indication of how much confidence the participant has in their knee [59] and will be administered as above. The distance will be measured from the toe of the starting position to the heel of the landing position. The farthest distance over three maximal attempts will be recorded. The 30-second knee bend test has been found to be reliable in meniscectomy patients (ICC 0.92 CI 95% 0.86-0.96) while variations of the one-leg rise test (ICC 0.84 CI 95% 0.69-0.92) and one-hop rise test (ICC 0.93 CI 95% 0.87-0.97) have also been found to be reliable in meniscectomy patients [58].

### Self-reported measures

Self-reported knee pain, other symptoms, ADL function, sport and recreation function and knee-related quality of life will be measured with the Knee Injury Osteoarthritis Outcome Score (KOOS). The KOOS consists of 42 items where each item has 5 possible answers (0–4), normalised to a score of 100, where lower score indicate worse symptoms. It has been shown to be valid and reliable in meniscectomised patients [60,61]. At the follow-up assessment, participants will rate their pain severity out of 10 on an 11-point numerical rating scale and their perceived change in physical function over the 12-week period (compared to baseline) on a seven-point ordinal scale (1-much worse to 7-much better). Also, at follow-up, participants will be questioned about their physiotherapy treatment experience.

### Other measures

A clinical measure of static knee alignment will be taken using an inclinometer [62]. Participants will be asked to stand comfortably, after which the tibial angle will be measured with respect to the vertical. This measure has been shown to be reliable (ICC = 0.94) and valid in relation to mechanical axis from full-length radiographs (r = 0.80) [62]. Physical activity will be measured using the Lower Extremity Activity Scale (LEAS), which will provide a global indication of physical activity level. From a series of options, participants select one option that best describes their current physical activity level. The LEAS is used in OA populations and has been found to be reliable and valid [63]. Participant adherence to the neuromuscular exercise program will be obtained by recording the number of physiotherapy sessions attended (out of a maximum number of eight). Participants will maintain a logbook to record the number of home exercises completed during the 12-week intervention period. For the purpose of this study, we will consider good adherence as: 1) at least 80% of physiotherapy sessions attended (i.e. 6 physiotherapy visits) and 2) at least 80% of the training sessions completed (i.e. 29 training sessions). Physiotherapists will also indicate their perceived impression of the participant’s overall adherence to the treatment on an 11-point numeric rating scale, with 0 representing ‘not at all and 10 being ‘completely as instructed’ [64].

### Sample size

A sample size of 62 participants ensures adequate power to detect significant changes in the external peak KAM and KAM impulse after the 12-week study period. As previously mentioned, a 14% reduction in peak KAM has been reported during one-leg rise in an uncontrolled study of people with knee OA following a neuromuscular exercise program [36]. Importantly, this reduction in peak KAM may be clinically relevant as it potentially corresponds to an approximate 2-fold reduction in risk of OA disease progression [25]. Therefore, we anticipate that a minimum 10% reduction in the peak KAM may be feasible with our neuromuscular program and that such a change may be potentially clinically meaningful. We anticipate that the peak KAM will remain unchanged in our control group participants. Based on our previous peak KAM data from APM participants [65], a 10% reduction equates to an absolute change of approximately 0.2 Nm/(BWxHT)%. Cautious estimates of the standard deviation of change scores are 0.3 Nm/(BWxHT)%, based on knee OA data from our laboratory investigating conservative treatments for knee OA [33]. Despite little being known about the clinically meaningful difference for KAM impulse or the response to interventions, we speculate a 10% change in KAM impulse is also feasible and potentially clinically meaningful following a neuromuscular intervention. We also anticipate that the KAM impulse will remain unchanged in our control group participants. Based on our previous KAM impulse data from APM participants [65], a 10% reduction equates to an absolute change of approximately 0.09 Nm.s/(BWxHT)%. Estimates of the standard deviation of change scores are 0.10 Nm.s/(BWxHT)% based on APM data from our laboratory where KAM impulse did not change over 2 years [65]. Therefore, a total of 27 participants per group is required to detect our anticipated changes in peak KAM and KAM impulse (assuming standard deviations as stated above) between the groups with 80% power (2 sided test, alpha=0.05). To account for 15% attrition, we will recruit 31 participants per group.

### Data and statistical analysis

Data analysis will be performed in a blinded manner and main comparative analyses between the two groups will be performed using an intention-to-treat analysis using all randomised participants. Multiple imputation methods will be used to address missing follow-up data. Participant characteristics and baseline data will be summarised by descriptive statistics. T-tests or non-parametric tests, as appropriate will be used to determine baseline comparability between the two groups. One-way ANOVA will be used to determine baseline comparability between the two groups. A non-parametric test will be used to determine baseline comparability for categorical data. For each dependent variable, change scores for each participant will be calculated by subtracting the follow-up score from the baseline score. Regression analyses adjusted for baseline values of the dependent variable and sex (the stratification variable) will be used to compare changes between baseline and follow-up for each independent variable. Standard diagnostic plots will check model assumptions. All tests will be two-tailed and performed at the 5% significance level.

### Timeline

Ethics approval was granted in April 2012 from the Human Research Ethics Committee of The University of Melbourne (HREC: 11377168) and Radiation Safety Human Services. The physiotherapists were recruited in February and trained in May 2012. Participant recruitment has commenced and it is expected that all participants will have completed the study by December 2013.

## Discussion

There is a need to assess the effect of neuromuscular exercise on knee joint loading [66]. Neuromuscular exercise is known to improve knee articular cartilage quality, physical function and patient-reported outcomes [43,59] demonstrating its potential benefits in rehabilitation post-APM. However, the effects of neuromuscular exercise on medial knee joint load in people at risk of developing knee OA or showing early signs of knee OA has yet to be rigorously determined. The peak external KAM was chosen as a primary outcome during normal pace walking for two reasons. Firstly, this parameter has been previously shown to be elevated post-APM relative to healthy controls [18] and secondly because of its association with medial compartment knee joint load and knee OA progression [20,25]. The peak external KAM during a one-leg rise is also included as a primary outcome considering it is a challenging task that pilot research has shown can be reduced following a neuromuscular training intervention [36]. Overall, two parameters of KAM are of interest, the peak KAM and KAM impulse during tasks of varying difficulty (walking, one-leg rise, one-leg hop for distance). These tasks were chosen to determine if medial knee joint load regardless of task can be reduced following this neuromuscular program. Due to the time demands on the participants for laboratory testing, muscle strength and muscle activity assessment will only be taken from the previously operated leg. Objective and self-report measures of function, as well as self-reported measures of symptoms will provide clinically relevant information and allow comparison with other studies. Overall, this study will comprehensively quantify the effects of neuromuscular exercise on biomechanical, functional and clinical measures associated with knee OA in a population at increased risk of developing knee OA or progressing early OA.

A major strength of this study is the pragmatic approach of treatment delivery by multiple community-based physiotherapists. This delivery approach ensures that findings will be reproducible and generalisable and if found effective, will provide sound evidence for a feasible APM rehabilitation option.

## Summary

This study uses a randomised controlled design to investigate the effect of a neuromuscular exercise program delivered by physiotherapists on medial knee joint load post-APM. The novel findings will enable evidence-base recommendations as to the effect of this conservative option for the reduction of medial knee joint loading in people considered at high risk to develop knee OA. Furthermore, findings will provide direction for future research into the effect of neuromuscular exercise on knee joint load.

## Competing interests

The authors have no competing interests to declare.

## Authors' contributions

KLB, RSH, EMR conceived the project and KLB and RSH are leading the co-ordination of the trial. KLB and PWH procured project funding while KLB, RSH, TVW, MH, EMR assisted with the protocol design. TVW and MH designed the biomechanical measures, while PWH designed the EMG measures. EMR, KLB, RSH, MH designed the neuromuscular exercise program and RSH and MH trained the physiotherapists. MS will lead the statistical analyses. All authors provided feedback on drafts of this manuscript and have read and approved the final paper.

## Pre-publication history

The pre-publication history for this paper can be accessed here:

http://www.biomedcentral.com/1471-2474/13/233/prepub
